# A systematic review of evidence-based clinical guidelines for vitamin D screening and supplementation over the last decade

**DOI:** 10.1186/s13690-025-01709-x

**Published:** 2025-08-29

**Authors:** Judit Zemp, Cigdem Erol, Estelle Kaiser, Carole E. Aubert, Nicolas Rodondi, Elisavet Moutzouri

**Affiliations:** 1 Institute of Primary Health Care (BIHAM), University of Bern, Bern, Switzerland; 2 Department of General Internal Medicine, Inselspital, Bern University Hospital, University of Bern, Bern, Switzerland

**Keywords:** Vitamin d, Guidelines

## Abstract

**Background:**

Amid growing evidence from observational data and trials with various results on the association between vitamin D and multiple diseases, numerous clinical guidelines were generated [1, 2]. The aims of this systematic review were to compare guidelines regarding recommendations for vitamin D screening and supplementation in the general adult population such as healthy people without pre-existing conditions or co-morbidities, but also for specific populations and find consensus for clinical practice.

**Methods:**

A systematic electronic search for clinical guidelines was conducted in the following databases: PubMed, Embase (Ovid), Cochrane Reviews and Google Scholar for the period from January 2013 to June 2024. Guidelines related to vitamin D screening and supplementation, targeted to the general adult population as well as to specific populations, released in Europe or North America in English, were included. Guidelines only aimed at children and adolescents, pregnant or breastfeeding women were excluded.

**Results:**

We identified 5853 records. After screening 92 full text studies, 31 guidelines were included for the final analysis. Two third of the guidelines recommended screening for people at risk for vitamin D deficiency, no guideline recommended screening for the general population. Almost one third recommended against any screening or did not specify, when screening would be appropriate. Half of the included guidelines recommended supplementation for people at risk, with varying definitions of people at risk. One third of these guidelines were aimed at people with osteoporosis and recommended vitamin D supplementation with varying doses, mainly with a dosage between 400 to 1000IU/day, one third recommended supplementation especially for the older population with a similar dosage, with varying age ranges for the definition of older people. There was no recommendation for supplementation for the general adult population without risk factors.

**Conclusions:**

Clinicians base their clinical practice on guidelines to improve and standardize the care for their patients. During our research we found many guidelines with very different recommendations for screening and for supplementation of vitamin D deficiency, so it was difficult to get a consensus. However, no guideline recommended screening or supplementation for the general adult population. No clear consensus could be reached for older people, people with osteoporosis or people with conditions increasing the risk for vitamin D deficiency, but most guidelines targeting these populations recommended supplementation with 400 to 1000IU/d and a vitamin D threshold with a minimum of 50 to 75 nmol/l. In that matter, further research is needed to get more conclusive data to get a better understanding of the effects of vitamin D deficiency and the benefit of a sufficient vitamin D level to generate standardized evidence-based recommendations in clinical guidelines, especially for the general population.

**Strengths and limitations of this research:**

The main strength of this systematic review is the robust search algorithm developed by experienced librarians. In addition, we applied clear and well-defined inclusion and exclusion criteria, focusing on the general adult population but also including specific populations, which enhanced the relevance of our findings to primary care practice. We used validated appraisal tools: the use of AGREE II ensured a structured and objective approach to assess guideline quality. The inclusion of guidelines up to mid-2024 makes the review current and highly relevant for clinical practice today. The findings may have potential implications for harmonizing international vitamin D recommendations and informing public health policy. Regarding the limitations, our analysis revealed that some guidelines neither contained clear recommendations, nor provided strong evidence, which made comparison between guidelines challenging. We excluded guidelines focusing on specific ethnicities outside of Europe or North America. While this may be considered a limitation, it was done with the understanding that certain geographic populations may have differing baseline vitamin D levels. We included guidelines, which referred to a systematic review conducted for this purpose. However, we did not assess the quality of each systematic review. Furthermore, the concept of evidence-based guidelines is complex, and it must be acknowledged that although a systematic review has been performed the included guidelines themselves may not be necessarily unequivocally evidence-based.

**Supplementary Information:**

The online version contains supplementary material available at 10.1186/s13690-025-01709-x.

**Table:** 

**Text box 1. Contributions to the literature**
• There is a rising number of vitamin D screening and supplementation especially in the general population, which is linked with high health care costs.
• Several guidelines regarding recommendations for vitamin D screening and supplementation were published over the last years both for the general population and for people at risk presenting huge differences in their recommendations.
• Guidelines did not recommend screening or supplementation of vitamin D in the general population because of weak evidence of benefit in trials.
• Screening of people at risk was recommended in some, but not in all guidelines, and there was no consensus for recommendations of supplementation regarding dosage and threshold of vitamin D, which may lead to uncertainty for clinicians.
• Further evaluation of clinical trials is needed to get more conclusive data for a better understanding of the effects of vitamin D deficiency and the benefit of a sufficient vitamin D level to generate standardized evidence-based recommendations in clinical guidelines, especially for the general population.

## Background

In recent decades, the role of vitamin D and its deficiency has attracted increased attention. Several observational studies have described the relevant role of vitamin D in musculoskeletal disorders, but also found associations of low 25-hydoxyvitamin D (25(OH)D) levels with an increased risk of many other diseases, including metabolic, cardiovascular, malignant, autoimmune, and infectious diseases [1, 2]. Although these associations are still being explored, there is an increasing rate of screening for vitamin D deficiency and supplementation with different formulations of vitamin D, which is linked with high health care costs [3, 4].

Vitamin D requirements can vary from person to person, depending on many different factors, therefore it is difficult to define an optimal serum level of 25(OH)D [1]. Because of growing evidence from observational studies, numerous trials have been conducted to assess the effect of vitamin D on a variety of diseases. In these trials, various vitamin D supplementation doses and administration schedules have been used while the enrolled participants differed regarding to baseline 25(OH)D levels [5]. This may explain the remaining uncertainty regarding health benefits of improving vitamin D levels.

Several recommendations for screening and supplementation in numerous vitamin D guidelines, especially in the general population, have been generated with partly huge differences [5]. Guidelines are commonly used in everyday clinical practice in order to standardize diagnostic and treatment protocols for optimized outcomes. However, several guidelines with differences in recommendations make implementation in clinical practice difficult. For example, both the US Preventive Service Task Force and the recently published guideline of the Endocrine Society recommended against any screening for vitamin D deficiency, but while the US Preventive Service Task Force recommended a supplementation just for people with low vitamin D without further specification, the Endocrine Society recommended a supplementation for a more wider target population [5–7].

The aims of this systematic review were to synthesize and compare the recommendations of various evidence-based guidelines regarding vitamin D screening and supplementation including both healthy people and specific populations such as older people, people at risk of falls and fractures, patients with osteoporosis and adults with other underlying conditions increasing the risk of vitamin D deficiency.

## Methods

Initially we formulated the following research question: “According to evidence-based guidelines in a European country and in North America, which adult population should be screened for vitamin D deficiency and/or treated with vitamin D supplementation, and what is the recommended dosage?“.

The systematic review aimed to compare clinical guidelines with recommendations for screening for vitamin D deficiency or for vitamin D supplementation from January 2013 up to June 2024. We set the limit to 2013 because guidelines are usually updated more frequently than 10 years.

For our research algorithm, we consulted an experienced health research librarian. We used the standardized search filters for identifying clinical practice guidelines developed and validated by the Canadian Agency for Drugs and Technologies in Health (CADTH), which can be used in databases. Those filters have demonstrated high sensitivity, and they can effectively retrieve relevant guidelines [8]. We conducted a systematic electronic search in PubMed, Embase (Ovid), Cochrane Reviews and Google Scholar. The search strategy is presented in the Appendix. We additionally searched in the following sources: Swiss Federal Office, US Preventive Service Task Force and National Institute for Health and Clinical Excellence (NICE). Two of three independent reviewers (EM, JZ, CE) [9] screened the retrieved papers based on the title and abstract using Rayyan, a free web application designed to help researchers with the screening process in systematic reviews [10]. Following inclusion criteria were defined: (a) guidelines that included recommendations on vitamin D screening and supplementation based on a systematic review (b) published in the defined time period in Europe or North America in English (c) referring to the adult population. Exclusion criteria were: (a) publication was not about vitamin D treatment or it only referred to dietary supplementation (b) publication did not address the adult population (c) publication was an old version of a more recently published guideline (d) full text was not available in English (e) publication focused on a population outside of Europe or North America (f) publication was specific to pregnant or lactating population. Then the reviewers assessed the full-text publications regarding inclusion or exclusion criteria, compared their results and discussed differences until consensus was reached.

One of two reviewers (JZ, CE) extracted and one (EM) controlled the following data: region and year of publication, institution, population referred to in the guidelines, screening and treatment recommendations, purpose of supplementation, 25(OH)D threshold for deficiency or insufficiency, conclusion and evidence of the study, gaps of the guidelines and research needs mentioned in the guidelines.

For quality appraisal (without grading the strength of evidence) we used AGREE II (Appraisal of Guidelines Research and Evaluation version 2) instrument, a well-established tool to evaluate the methodological rigor and transparency through which a guideline is developed [11].

For reporting of the present review, we followed the PRISMA guidelines [12].

## Results

**Fig. 1:**
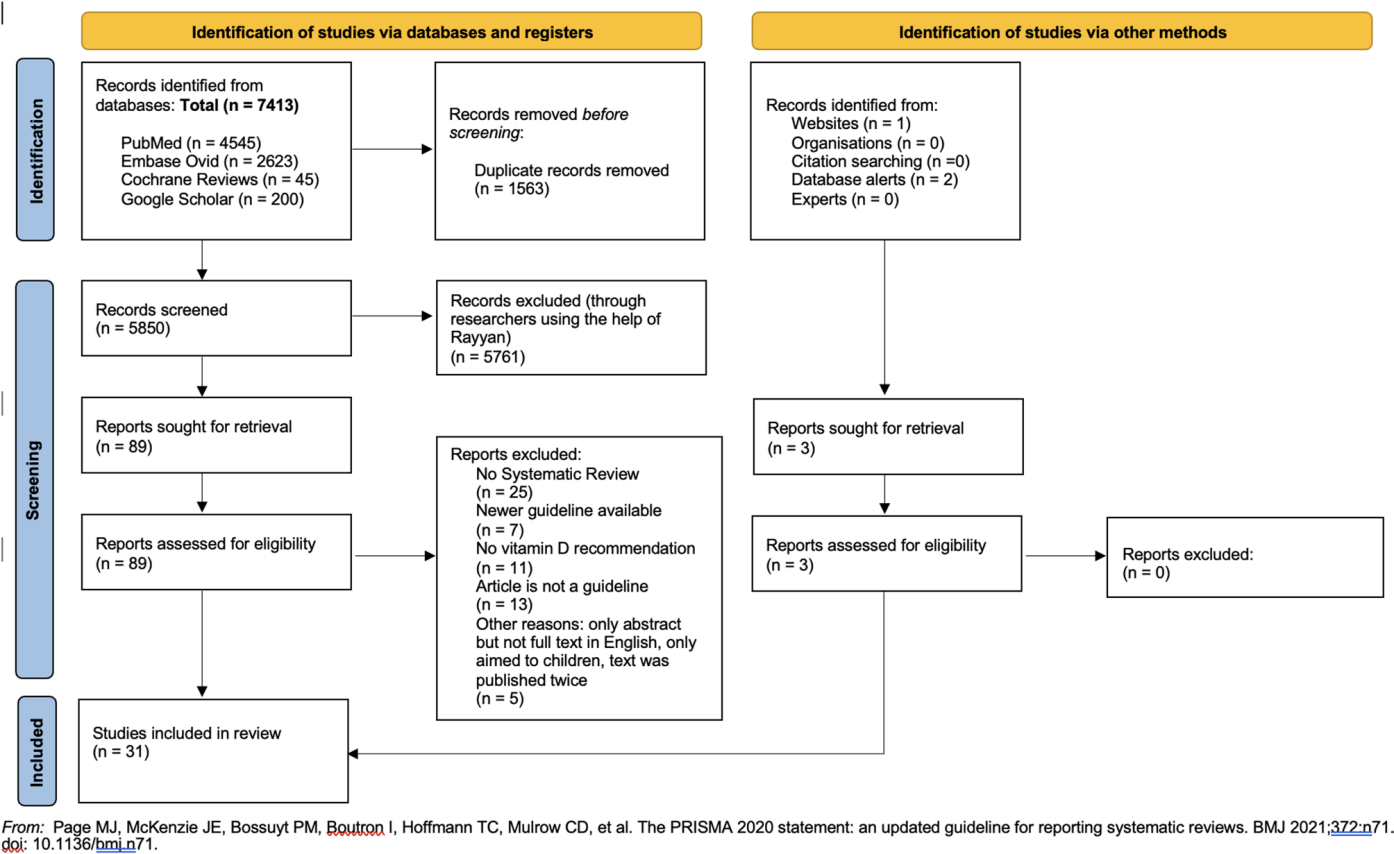
Searching strategy for vitamin D guidelines

**Fig. 2:**
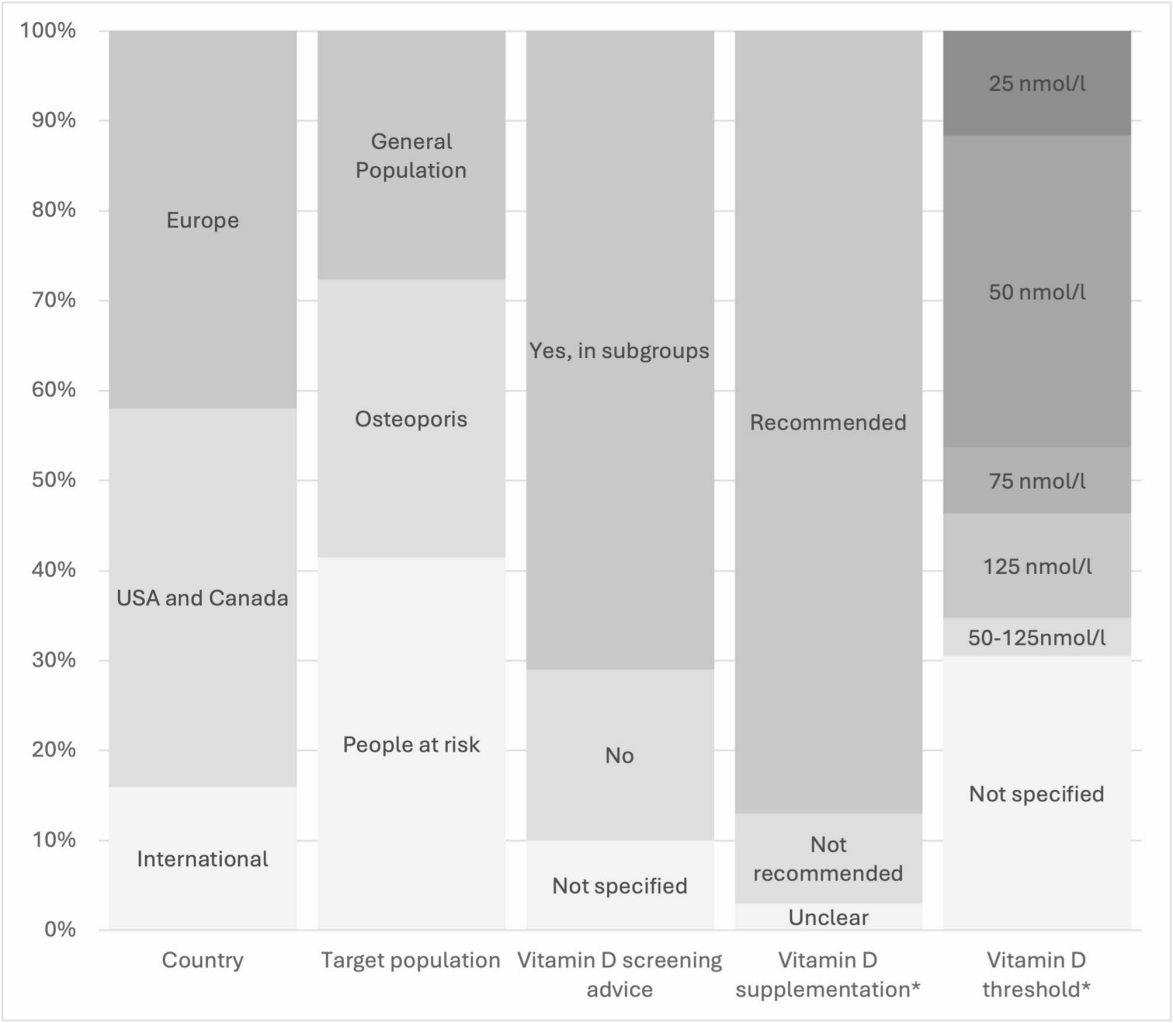
Overview of characteristics and results for recommendations of the included guidelines from January 2013 up to June 2024. *only *N* = 27 from 31 guidelines gave recommendations

We identified 5853 records of which 92 full texts were screened and 31 guidelines retained for analysis (Fig. 1; Table 1 (summary); Appendix Table 2 (full analysis)). Overview of characteristics and results of recommendations are presented in Fig. 2. Excluded guidelines, which full text was screened, are presented in Appendix Table 3.

**Table 1:** Overview of included guidelines regarding recommendations for screening for vitamin D deficiency or for vitamin D supplementation from January 2013 up to June 2024 (Summary)

Guideline name	Country/ Year	Screening recommended	Treatment recommended	25-OH-D threshold [nmol/l]
Vitamin D supplementation in elderly or postmenopausal women: a 2013 update of the 2008 recommendations, European society for clinical and economic aspects of osteoporosis and osteoarthritis (ESCEO)	Europe, 2013	Yes: people at risk	800–1000 IU/d	50–75
Vitamin D - a systematic literature review for the 5th edition of the Nordic Nutrition Recommendations, Nordic Nutrition	Finland, Norway, Iceland, 2013	Not recommended	800 IU/d	50
Recommendations Abstracted from the American Geriatrics Society Consensus Statement on Vitamin D for Prevention of Falls and Their Consequences, American geriatrics	USA, 2014	Yes: people at risk	All adults ≥ 65y: 1000IU/d	75
Celiac disease: diagnosis and treatment, Danish Society for Gastroenterology	Denmark, 2014	Yes: All with untreated celiac disease	Yes, but not specified	Not specified
European Society of Endocrinology clinical guideline: Treatment of chronic hypoparathyroidism in adults	Europe, 2015	Not specified but recommended adequate vitamin D status: Indirectly, all with chronic hypoparathyroidism	400– 800 IU/d to patients treated with activated vitamin D analogues	50
Lack of Evidence linking Calcium with or without Vitamin D Supplementation to Cardiovascular Disease in generally healthy Adults: A clinical Guideline from the National Osteoporosis Foundation and the American Society for Preventive Cardiology	USA, 2016	Not recommended	Not specified	Not specified
Vitamin D: supplement use in specific population groups, NICE	UK, 2017	Yes: people with symptoms of deficiency or at very high risk	Yes, but not specified	<25
Treatment of low bone density or osteoporosis to prevent fractures in men and women: A clinical practice guideline update from the American college of physicians	USA, 2017	Not specified	Yes, but not specified	Not specified
Diagnosis, Evaluation, Prevention, and Treatment of Chronic Kidney Disease-Mineral and Bone Disorder: Synopsis of the kidney disease: Improving Global Outcomes 2017 Clinical Practice Guideline Update, KDIGO	International, 2018	Yes: Only patients with levels of intact PTH progressively rising or persistently above the upper normal limit	Yes, but not specified	Not specified
Vitamin D, calcium, or combined supplementation for the primary prevention of fractures in community-dwelling adults, recommendation statement, US Preventive Services Task Force	USA, 2018	Not specified	Not recommended	Not specified
Current Vitamin D status in European and Middle East countries and strategies to prevent Vitamin D deficiency: a position statement of the European Calcified Tissue Society	Europe, 2019	Yes: people at risk	400–800 IU/d	50
British Society of Gastroenterology consensus guidelines on the management of inflammatory bowel disease in adults	UK, 2019	Yes: people with Crohn’s disease and ulcerative colitis	800 IU/d	Not specified
Clinical practice recommendations for the diagnosis and management of X-linked hypophosphatemia	International, 2019	Yes: Children and adults with X-linked hypophosphatemia (XLH) and any first-generation family member of a patient with XLH should be investigated	Yes: Dosage should be adjusted to keep PTH levels within the normal range (10-65pg/ml); range for Calcitriol 0.5-0.75mcg/d	Not specified Dose can be adjusted based on serum levels of PTH and urinary calcium excretion.
Vitamin D and bone health: a practical clinical guideline for patient management, Royal Osteoporosis Society	Europe, 2020	Yes: people with symptoms of bone diseases	< 30nmol/l): high dose treatment initially, then long-term maintenance treatment 30–50 nmol/l: over the counter dose of 800– 2000UI/d, long term. People at risk: 400UI/d	50
Vitamin D testing, Swiss Federal Office of Public Health (FOPH)	Switzerland, 2020	Yes: people at risk	600–800 IU/d	50
American Association of Clinical Endocrinologists/ American College of Endocrinology Clinical Practice Guidelines for the Diagnosis and Treatment of Postmenopausal Osteoporosis-Update	USA, 2020	Yes: people at risk	1000–2000 IU/d	30–50
The Belgian Bone Club 2020 guidelines for the management of osteoporosis in postmenopausal women	Belgia, 2020	Yes: women at risk	800 − 1000 IU/d.	50
Executive Summary of the Academy of Nutrition and Dietetics and the National Kidney Foundation Clinical Practice Guideline for Nutrition in CKD	USA, 2020	Not specified	Yes, but not specified	Not specified
Recommendations Based on Evidence by the Andalusian Group for Nutrition Reflection and Investigation (GARIN) for the Pre- and Postoperative Management of Patients undergoing Obesity Surgery	Europe, 2020	Yes: All patients undergoing bariatric surgery	Preoperatively with a goal of > 50 nmol/L of 25(OH)D levels, postoperatively: 880 IU/d biliopancreatic diversion/Scopinaro surgeries: 2000 IU/d	50
Osteoporosis clinical guideline for prevention and treatment executive summary, National Osteoporosis Guideline Group	Europe, 2021	Yes: Suggested for the investigation of osteoporosis/fragility fractures	800IU/d	Not specified
Screening for Vitamin D, US Preventive Service Task Force	USA, 2021	Not recommended	Yes, but not specified	Not specified
Congress of Neurological Surgeons Systematic Review and Evidence-Based Guidelines for Perioperative Spine: Preoperative Osteoporosis Assessment	USA, 2021	Yes: preoperative osteoporosis assessment	Yes, but not specified	50
Chronic Obstructive Pulmonary Disease: A 2019 Evidence Analysis Centre Evidence- Based Practice, The academy of Nutrition and dietetics	USA, 2021	Yes: COPD patients	Yes, but not specified	25
Vitamin, Mineral, and Multivitamin Supplementation to Prevent Cardiovascular Disease and Cancer, US Preventive Services Task Force Recommendation Statement	USA, 2022	Not recommended	Not recommended	Not specified
Guideline No. 422 g: Menopause and Osteoporosis, Society of Gynaecologists of Canada	Canada, 2022	Yes: all adults > 65y and all postmenopausal women	800–2000 IU/d	75–125
Definition, Assessment, and Management of Vitamin D Inadequacy: Suggestions, Recommendations, and Warnings from the Italian Society for Osteoporosis, Mineral Metabolism and Bone Diseases (SIOMMMS)	Italy, 2022	Yes: people at risk	800–2000 IU/d	50–125 in general population; 75–125 in population at risk
Evaluation and Management of Hypoparathyroidism Summary Statement and Guidelines from the Second International Workshop	International, 2022	Yes: all patients with Hypoparathyroidism	Cholecalciferol: 1000-100000IU/d based on 25(OH)D level Calcitriol: 0.25-3mcg/d administered in divided doses	75–125
2022 American College of Rheumatology Guideline for the Prevention and Treatment of Glucocorticoid-Induced Osteoporosis	USA, 2023	Yes: patients > 40y with GC treatment for > 3months	600–800 IU/d or more	75–125
Vitamin D status and supplementation before and after Bariatric Surgery: Recommendations based on a systematic review and meta-analysis	International, 2023	Yes: all bariatric patients, pre- and post-surgery	2000 IU/d	75
2023 AHA/ACC/ACCP/ASPC/NLA/PCNA Guideline for the Management of Patients with Chronic Coronary Disease: A Report of the American Heart Association / American College of Cardiology Joint Committee on Clinical Practice Guidelines	USA, 2023	Not recommended	Not recommended	Not specified
Vitamin D for the Prevention of Disease: An Endocrine Society Clinical Practice Guideline	International, 2024	Not recommended	Children and adolescents aged 1 to 18 years (300-2000IU/day), general population > 75 years (400-3333IU/day), pregnant women (600-5000IU/day), adults with high-risk prediabetes (842-7543IU/day) and adults > 50 years who have indications for vitamin D supplementation or treatment (dosage not specified).	Not specified

### Characteristics

Among the included guidelines there were 42% (13/31) from Europe, 42% (13/31) from North America (United States and Canada), and 16% (5/31) were international guidelines (Appendix Table 4). Ten guidelines (10/31) were focused on the general population, nine (9/31) on people with osteoporosis, and 12 (12/31) on special populations like people with chronic kidney disease, hypoparathyroidism, X-linked hypophosphatemia, inflammatory bowel disease, obesity, cardiovascular disease, or COPD (chronic obstructive pulmonary disease). Older people were mentioned as a special subpopulation in these guidelines (10/31). The latest guideline from the Endocrine Society only referred to the general population without risk factors for vitamin D deficiency, like chronic liver or kidney diseases, bone diseases, obesity, malabsorption syndromes, medication with effects on the vitamin D metabolism, or low sun exposure, only older people were also included. All the other guidelines referred to general population also included these people at risk.

### Recommendations on screening

Two third (22/31) of the guidelines recommended screening for people at risk for vitamin D deficiency (12/31), with symptoms, with osteoporosis or with high risk for development of osteoporosis (10/31). There was no guideline recommending screening for the general population. Nine guidelines recommended against any screening or did not specify, when screening would be appropriate [5–7, 13–18].

### Recommendations on supplementation

In total, there were four guidelines, which did not clearly recommend vitamin D supplementation [14] or recommend against supplementation [7, 13, 15] because of lack of benefits. All other included guidelines recommended vitamin D supplementation (27/31), but with differences in doses ranged from 400 to 3000IU for daily supplementation with cholecalciferol (Table 1, Appendix Table 2). Nearly one third (9/31) of these guidelines were aimed to people with osteoporosis [17, 19–26]one third (9/31) recommended supplementation for the older population (age range > 65-75years) [5, 19–21, 27–31] and over half of these guidelines (18/31) recommended supplementation for people at risk for vitamin D deficiency including dark skinned people, chronic kidney or liver disease, autoimmune diseases, prediabetes, following bariatric surgery and with malabsorption syndromes, or people taking glucocorticoid or other medicine with effects on vitamin D and bone mineral metabolism [5, 16, 18, 22, 27–40]. Five guidelines recommended a supplementation of 400 to 800IU/d [21, 22, 27, 31, 33]six guidelines recommended 800 to 1000IU/d [16, 20, 23, 24, 28, 35]five guidelines recommended up to 2000IU/d [19, 25, 30, 37, 39]one guideline defined different doses for different subpopulations with ranges between 400 to 3000IU/d [5]and one third (10/31) did not define a dose and recommended an individual evaluation based on vitamin D levels, PTH levels (in case of X-linked hypophosphatemia), calcium levels, or symptoms of hypercalciuria. To sum up, supplementation mainly with a dosage between 400 to 1000IU/d was recommended for patients with manifest osteoporosis, for people with conditions increasing the risk of vitamin D deficiency and for older people. There was no recommendation for supplementation for the general adult population without risk factors.

### Recommendations on optimal 25(OH)D thresholds

The definition of an adequate 25(OH)D threshold to reach a sufficient level for bone health was very inconsistent in different guidelines and ranged from 25 to 125 nmol/l. Three guidelines aimed 25(OH)D threshold above 25nmol/l [25, 29, 38]nearly one third recommended levels above 50nmol/l [16, 20, 21, 23, 26, 27, 31, 33, 37]two guidelines recommended levels above 75nmol/l [28, 39]and three guidelines recommended levels up to 125nmol/l [19, 22, 40]. One guideline recommended vitamin D levels above 50nmol/l up to 125nmol/l for the general population and 75nmol/l up to 125nmol/l for people at risk [30]. Nearly one third of the guidelines did not specified an optimal 25(OH)D threshold [5, 7, 17, 18, 24, 32, 34–36].

### Recommendations for older people

One third (10/31) of the included guidelines aimed for adults aged > 65-75years and recommended supplementation with a dosage between 400 to 1000IU/d to prevent osteoporosis and to reduce the risk of falls and fractures. The recommended 25(OH)D threshold for a sufficient level differed from 25 to 125nmol/l.

### Results on quality

Using the AGREE II instrument we get an overview of quality of the included guidelines concerning for example patient enrollment, practice advise and transparency during development (Appendix Table 5). Many guidelines were limited in methodological rigor, for example they did not clearly describe criteria for selection of evidence or the methods for formulating the recommendations. We found also a lack in clarity of presentation, for example some guidelines did not accurately describe treatment recommendation or defined vitamin D levels which should be reached.

## Discussion

With this systematic review, we compared 31 evidence-based guidelines regarding recommendations for vitamin D screening and supplementation, both for the general adult population and specific populations like older persons, people with osteoporosis or people at risk for vitamin D deficiency and secondary bone disease. Compared to other systematic reviews of clinical guidelines concerning vitamin D recommendations [41]the present review did not focus on one special population like people with osteoporosis or the general population but searched for the whole spectrum in current guidelines up to June 2024 concerning vitamin D screening and supplementation. One third of the included guidelines focused on the general population, one third on people with osteoporosis and the rest on different subpopulations, but mainly with risk of developing a secondary osteoporosis.

Comparing the 31 included guidelines, we found big differences in the recommendations for screening. Regarding the general population, the evidence for screening for vitamin D deficiency is weak [42]. In support of this, no guideline recommended screening for the general population. This was unexpected considering the increasing rates of vitamin D screening during the last years, especially in the general population. Regarding people at risk for vitamin D deficiency, people with osteoporosis or older people, two third of the guidelines recommended screening for vitamin D, like the Swiss Federal Office or the European Calcified Tissue Society [27, 31]. Guidelines like those of the US Preventive Service Task Force or lately the Endocrine Society recommended against any screening [5, 6] including the general population, people with underlying conditions increasing the risk for vitamin D deficiency, people with osteoporosis or older people. Reasons for recommendations against any screening were lack of benefit in clinical trials and high associated costs. But also lack of standardized laboratory testing of vitamin D levels is an important factor that should gain more attention regarding definition of vitamin D deficiency and for interpretation of measurement [43]. Different methods for measurement (either antibody-based methods or liquid chromatography-mass spectrometry), different reference material, inconsistent reference values and at least using different unit of measure should be considered [44].

The available clinical evidence did not clearly support routine vitamin D supplementation in the general adult population regardless of baseline 25(OH)D levels. Therefore, the guidelines did not recommend supplementation in the general population. Most guidelines recommended supplementation with vitamin D only for people at risk for vitamin D deficiency, people with osteoporosis or risk of developing osteoporosis or for older people. It needs to be mentioned that the definition of “older people” ranges between an age of > 65 to > 75 years in the included guidelines. The range of recommended daily supplementation of vitamin D and optimal serum 25(OH)D levels also varied considerably. Most guidelines recommended a vitamin D supplementation of at least 400 IU/d cholecalciferol for bone health and prevention of osteoporosis, the majority still recommended the mainly used dosage of 800 to 1000IU/d (Table 1, Appendix Table 2). For example, guidelines aimed at people with osteoporosis recommended a supplementation with 400 to 1000IU/d, but also other guidelines targeting other populations recommended the same dosage, indicating that there is no unified consensus limited to a single group. However, nearly one-third of the analyzed guidelines did not specify a dosage, instead they recommended an individually adapted supplementation based on different factors like serum levels of vitamin D, calcium or parathyroid hormone (PTH). Guidelines were consistent regarding the recommendation for daily or weekly intake of vitamin D. Daily low-dose vitamin D regimes (daily dosage usually between 400 to 2000IU/d) reduce the risk of falling, especially in older people, compared with periodic doses like monthly applications, that may increase it [45]while high doses (dosage administered every 3 to 12 months with 250000 to 1000000IU [23] or a daily dosage about 4000IU [46]) have been associated with an increased risk of falls and other adverse effects [41, 46–50]. Until now no data are available regarding whether vitamin D supplementation should be administered indefinitely [30]so there is no evidence-based recommendation for the duration of vitamin D supplementation. It is suggested that a supplementation should be maintained, until the cause of vitamin D deficiency has been removed, if possible. One guideline suggested that the vitamin D level should be improved step by step by using a simple, regular, and effective supplementation with suitable and safe dosages to prevent and treat vitamin D deficiency [46]. Some guidelines defined a safe dosage with daily low-dose vitamin D between 400 to 2000IU/d [45, 46]. Besides supplementation an adequate diet, lifestyle modifications and sun exposure were recommended [5, 22, 46]. At least three guidelines recommended against supplementation because of lack of benefit [7, 13, 15]but they aimed at only a special subpopulation (people with coronary diseases).

Regarding vitamin D threshold, different guidelines defined different values for an ideal 25(OH)D level. In 2011 the Institute of Medicine (IOM) defined deficiency of 25(OH)D as a serum value < 12ng/ml (30nmol/l), insufficiency as 12-20ng/ml (30-50nmol/l), and sufficiency as 20-30ng/ml (50-75nmol/l), respectively [51]. For clinicians there is a wide zone of uncertainty between 12ng/ml (30nmol/l) and 30ng/ml (75nmol/l) [52]. As a matter of fact, this was reflected in the different recommendations of the guidelines regarding the optimal 25(OH)D threshold, which ranged from 25 to 125 nmol/l (Table 1). Half of the guidelines (16/31) recommended a minimum of 50 to 75 nmol/l. We also found very different recommendations for vitamin D threshold for the same population, for example the cutoff point for people with osteoporosis ranged from 50nmol/l ^[23],36^ to 75-125nmol/l [19, 22]. The Endocrine Society did not confirm its previously proposed definitions of vitamin D sufficiency or insufficiency in its latest guideline, because available clinical trial evidence does not clearly support that [5, 52].

Clinicians rely on clinical practice on guidelines to improve and standardize care while simultaneously ensuring an evidence-based approach. Ideally, there should be little differences between practical guidelines to get an international standard. However, in this work, we found many guidelines with very different content and inconsistent recommendations for screening and supplementation of vitamin D deficiency. The analysis showed weakness in rigor of development and presentation of the recommendations. Therefore, decision-making could be difficult for clinicians and may hinder implementation in practice. A solution could be the discussion of the different recommendations in quality circles, which are a useful instrument in primary care settings for critical thinking including the use of clinical guidelines. In addition, the concept of shared decision-making between clinicians and their patients could be useful to discuss the different recommendations of the guidelines as well as pros and cons for screening and supplementation of vitamin D. More research using individual participant data from available trials with focus on those participants with truly low vitamin D levels may be needed to determine the cutoff for the definition of vitamin D deficiency and insufficiency, to examine the benefits of vitamin D especially for its extra skeletal effects and all-cause mortality, and to create more consistent recommendations for screening and supplementation of vitamin D.

## Conclusion

In this systematic review, we identified many guidelines with very different recommendations for screening and for supplementation of vitamin D deficiency. Comparison between the guidelines, even for the same population, was difficult and it was tough to get a consensus. However, we have found no recommendation for screening or supplementation for the general adult population. But for older people, people with osteoporosis or people with conditions increasing the risk for vitamin D deficiency no clear consensus could be reached. Most guidelines targeted to these populations recommended supplementation with 400 to 1000IU/d and a vitamin D threshold with a minimum of 50 to 75 nmol/l. Using these different guidelines in daily practice may result in unclarity for the clinicians. Further research is needed to get a better understanding of the effects of vitamin D deficiency and the benefit of a sufficient vitamin D level. Engaging international and national health organizations to collaborate to evaluate the available data from multiple clinical trials by making individual data available as well as creating unified evidence-based guidelines with less differences in recommendations may be a solution.

## Supplementary Information

Below is the link to the electronic supplementary material.

Supplementary Material 1

## Data Availability

No datasets were generated or analysed during the current study.
