# Supplementary material for: A systematic review of evidence-based clinical guidelines for vitamin D screening and supplementation over the last decade
Source: Arch Public Health. 2025 Aug 29;83:221. doi: 10.1186/s13690-025-01709-x (PMC12395781; doi:10.1186/s13690-025-01709-x)
Supplement: Supplementary file 1 — Supplementary Material 1 [file 13690_2025_1709_MOESM1_ESM.docx]

**Appendix**

**Overview databases and results** (Date last searched: **21 May 2024**)

**Full Search Strategy per Database**

| **Database searched** | **Platform** | **Years of coverage** | **Records** | **Records after duplicates removed** |
| --- | --- | --- | --- | --- |
| PubMed | NLM | 1949 - Present | 4545 |  |
| Embase | Ovid | 1974 - Present | 2623 |  |
| Cochrane Reviews | Wiley | 1992 - Present | 45 |  |
| Google Scholar (search engine): relevance ranking top 200 200 | | | | |
| **Total** | | | 7413 | **5850** |

**Database limits applied: January 1, 2013 - December 31, 2024**

**Totally, 1563 duplicates were removed** using Deduklick^[1]^[1] Borissov, N., Haas, Q., Minder, B. et al. Reducing systematic review burden using Deduklick: a novel, automated, reliable, and explainable deduplication algorithm to foster medical research. Syst Rev 11, 172 (2022). https://doi.org/10.1186/s13643-022-02045-9

**Detailed search strategies per database**

**PubMed** concept 1 AND concept 2, NOT concept 3

| **1) Vitamin D** |
| --- |
| "Vitamin D"[Majr] OR "Vitamin D Deficiency"[Majr] OR vitamin d*[tiab] OR vitamin d2[tiab] OR vitamin d3[tiab] OR hydroxyvitamin d*[tiab] OR hydroxy-vitamin d*[tiab] OR hydroxylvitamin d*[tiab] OR alfacalcidol*[tiab] OR alphacalcidol*[tiab] OR calcamine[tiab] OR calciferol*[tiab] OR calciol*[tiab] OR calcidiol*[tiab] OR calcitriol*[tiab] OR calcifediol*[tiab] OR calciferol*[tiab] OR calcipotriene[tiab] OR calcipotriol*[tiab] OR cholecalciferol*[tiab] OR colecalcifer*[tiab] OR dehydrocholesterol*[tiab] OR dihydroxyvitamin d*[tiab] OR dihydroxy-vitamin d*[tiab] OR dihydroxycholecalciferol*[tiab] OR dihydroxy-cholecalciferol*[tiab] OR dihydroxycalciferol*[tiab] OR dihydroxy calciferol*[tiab] OR dihydroxycolecalciferol*[tiab] OR dihydroxyergocalciferol*[tiab] OR dihydroxy ergocalciferol*[tiab] OR doxercalciferol*[tiab] OR dihydrotachysterol*[tiab] OR dihydroercalciol*[tiab] OR dihydrotachysterin[tiab] OR doxercalciferol*[tiab] OR epicalcitriol*[tiab] OR ercalcidiol*[tiab] OR ercalcitriol*[tiab] OR ergocalciferol*[tiab] OR hydroxycholecalciferol*[tiab] OR hydroxycalciferol*[tiab] OR hydroxycolecalciferol*[tiab] OR hydroxyergocalciferol*[tiab] OR oxacalcitriol*[tiab] OR oxavitamin*[tiab] OR paricalcitol*[tiab] OR seocalcitol[tiab] OR tacalcitol[tiab] OR "d hypovitaminosis"[tiab] OR "hypo-vitaminosis d"[tiab] OR "hypovitaminosis d"[tiab] OR "d vitamin deficien*"[tiab] OR "vit* d deficien*"[tiab] OR "avitaminosis D"[tiab] OR "D avitaminosis"[tiab] OR nutritional supplement*[tiab] OR nutrition supplement*[tiab] OR nutrient supplement*[tiab] OR nutrients supplement*[tiab] OR dietary supplement*[tiab] OR diet supplement*[tiab] |
| **2) Guidelines** Search filter: *Guidelines -Broad -PubMed*. In: **CADTH Search Filters Database**. Ottawa: Canadian Agency for Drugs and Technologies in Health (CADTH); April 2, 2020. Available from: <https://searchfilters.cadth.ca/link/75> |
| "Clinical protocols"[MESH] OR "Consensus"[MESH] OR "Consensus development conferences as topic"[MESH] OR "Critical pathways"[MESH] OR "Guidelines as topic"[Mesh:NoExp] OR "Practice guidelines as topic"[MESH] OR "Health planning guidelines"[MESH] OR "Clinical Decision Rules"[MESH] OR "guideline"[pt] OR "practice guideline"[pt] OR "consensus development conference"[pt] OR "consensus development conference, NIH"[pt] OR position statement*[tiab] OR policy statement*[tiab] OR practice parameter*[tiab] OR best practice*[tiab] OR standards[ti] OR guideline[ti] OR guidelines[ti] OR standards[ot] OR guideline[ot] OR guidelines[ot] OR guideline*[cn] OR standards[cn] OR consensus*[cn] OR recommendat*[cn] OR practice guideline*[tiab] OR treatment guideline*[tiab] OR CPG[tiab] OR CPGs[tiab] OR clinical guideline*[tiab] OR guideline recommendation*[tiab] OR consensus*[tiab] OR ((critical[tiab] OR clinical[tiab] OR practice[tiab]) AND (path[tiab] OR paths[tiab] OR pathway[tiab] OR pathways[tiab] OR protocol*[tiab] OR bulletin[tiab] OR bulletins[tiab])) OR recommendat*[ti] OR recommendat*[ot] OR (care[tiab] AND (standard[tiab] OR path[tiab] OR paths[tiab] OR pathway[tiab] OR pathways[tiab] OR map[tiab] OR maps[tiab] OR plan[tiab] OR plans[tiab])) OR (algorithm*[tiab] AND (screening[tiab] OR examination[tiab] OR test[tiab] OR tested[tiab] OR testing[tiab] OR assessment*[tiab] OR diagnosis[tiab] OR diagnoses[tiab] OR diagnosed[tiab] OR diagnosing[tiab])) OR (algorithm*[tiab] AND (pharmacotherap*[tiab] OR chemotherap*[tiab] OR chemotreatment*[tiab] OR therap*[tiab] OR treatment*[tiab] OR intervention*[tiab])) |
| **3) Limits applied:**  The search was limited to studies involving adult human populations by excluding records indexed with terms for the pediatric population and animal studies. Publication types such as letters, editorials, and notes were also excluded.  **Time period:**  January 2013 to December 2024 |
| NOT (("infant"[mesh] OR "child"[mesh] OR "adolescent"[mesh]) NOT "adult"[mesh]) NOT ("animals"[mesh] NOT "humans"[mesh]) NOT (letter[pt] OR news[pt] OR comment[pt] OR editorial[pt]) NOT ((infant*[ti] OR child*[ti] OR newborn*[ti] OR pediatric*[ti] OR paediatric*[ti] OR juvenile[ti]) NOT adult[ti])  Filters applied: from 2013/1/1 - 2024/12/31 |

**PubMed Search** string for replication:

(("Vitamin D"[Majr] OR "Vitamin D Deficiency"[Majr] OR vitamin d*[tiab] OR vitamin d2[tiab] OR vitamin d3[tiab] OR hydroxyvitamin d*[tiab] OR hydroxy-vitamin d*[tiab] OR hydroxylvitamin d*[tiab] OR alfacalcidol*[tiab] OR alphacalcidol*[tiab] OR calcamine[tiab] OR calciferol*[tiab] OR calciol*[tiab] OR calcidiol*[tiab] OR calcitriol*[tiab] OR calcifediol*[tiab] OR calciferol*[tiab] OR calcipotriene[tiab] OR calcipotriol*[tiab] OR cholecalciferol*[tiab] OR colecalcifer*[tiab] OR dehydrocholesterol*[tiab] OR dihydroxyvitamin d*[tiab] OR dihydroxy-vitamin d*[tiab] OR dihydroxycholecalciferol*[tiab] OR dihydroxy-cholecalciferol*[tiab] OR dihydroxycalciferol*[tiab] OR dihydroxy calciferol*[tiab] OR dihydroxycolecalciferol*[tiab] OR dihydroxyergocalciferol*[tiab] OR dihydroxy ergocalciferol*[tiab] OR doxercalciferol*[tiab] OR dihydrotachysterol*[tiab] OR dihydroercalciol*[tiab] OR dihydrotachysterin[tiab] OR doxercalciferol*[tiab] OR epicalcitriol*[tiab] OR ercalcidiol*[tiab] OR ercalcitriol*[tiab] OR ergocalciferol*[tiab] OR hydroxycholecalciferol*[tiab] OR hydroxycalciferol*[tiab] OR hydroxycolecalciferol*[tiab] OR hydroxyergocalciferol*[tiab] OR oxacalcitriol*[tiab] OR oxavitamin*[tiab] OR paricalcitol*[tiab] OR seocalcitol[tiab] OR tacalcitol[tiab] OR "d hypovitaminosis"[tiab] OR "hypo-vitaminosis d"[tiab] OR "hypovitaminosis d"[tiab] OR "d vitamin deficien*"[tiab] OR "vitamin-d-deficien*"[tiab] OR "vitamine-d-deficien*"[tiab] OR "vit-d-deficien*"[tiab] OR "avitaminosis D"[tiab] OR "D avitaminosis"[tiab] OR nutritional supplement*[tiab] OR nutrition supplement*[tiab] OR nutrient supplement*[tiab] OR nutrients supplement*[tiab] OR dietary supplement*[tiab] OR diet supplement*[tiab]) AND ("Clinical protocols"[MESH] OR "Consensus"[MESH] OR "Consensus development conferences as topic"[MESH] OR "Critical pathways"[MESH] OR "Guidelines as topic"[Mesh:NoExp] OR "Practice guidelines as topic"[MESH] OR "Health planning guidelines"[MESH] OR "Clinical Decision Rules"[MESH] OR "guideline"[pt] OR "practice guideline"[pt] OR "consensus development conference"[pt] OR "consensus development conference, NIH"[pt] OR position statement*[tiab] OR policy statement*[tiab] OR practice parameter*[tiab] OR best practice*[tiab] OR standards[ti] OR guideline[ti] OR guidelines[ti] OR standards[ot] OR guideline[ot] OR guidelines[ot] OR guideline*[cn] OR standards[cn] OR consensus*[cn] OR recommendat*[cn] OR practice guideline*[tiab] OR treatment guideline*[tiab] OR CPG[tiab] OR CPGs[tiab] OR clinical guideline*[tiab] OR guideline recommendation*[tiab] OR consensus*[tiab] OR ((critical[tiab] OR clinical[tiab] OR practice[tiab]) AND (path[tiab] OR paths[tiab] OR pathway[tiab] OR pathways[tiab] OR protocol*[tiab] OR bulletin[tiab] OR bulletins[tiab])) OR recommendat*[ti] OR recommendat*[ot] OR (care[tiab] AND (standard[tiab] OR path[tiab] OR paths[tiab] OR pathway[tiab] OR pathways[tiab] OR map[tiab] OR maps[tiab] OR plan[tiab] OR plans[tiab])) OR (algorithm*[tiab] AND (screening[tiab] OR examination[tiab] OR test[tiab] OR tested[tiab] OR testing[tiab] OR assessment*[tiab] OR diagnosis[tiab] OR diagnoses[tiab] OR diagnosed[tiab] OR diagnosing[tiab])) OR (algorithm*[tiab] AND (pharmacotherap*[tiab] OR chemotherap*[tiab] OR chemotreatment*[tiab] OR therap*[tiab] OR treatment*[tiab] OR intervention*[tiab])))) NOT (("infant"[mesh] OR "child"[mesh] OR "adolescent"[mesh]) NOT "adult"[mesh]) NOT ("animals"[mesh] NOT "humans"[mesh]) NOT (letter[pt] OR news[pt] OR comment[pt] OR editorial[pt]) NOT ((infant*[ti] OR child*[ti] OR newborn*[ti] OR pediatric*[ti] OR paediatric*[ti] OR juvenile[ti]) NOT adult[ti]) AND (2013/1/1:2024/12/31[pdat])

**Embase (Ovid)**

| # | Search query |
| --- | --- |
| 1 | clinical pathway/ |
| 2 | exp clinical protocol/ |
| 3 | exp consensus/ |
| 4 | exp consensus development conference/ |
| 5 | exp consensus development conferences as topic/ |
| 6 | clinical pathway/ |
| 7 | guidelines as topic/ |
| 8 | exp practice guideline/ |
| 9 | practice guidelines as topic/ |
| 10 | health planning guidelines/ |
| 11 | health care planning/ |
| 12 | clinical decision rule/ |
| 13 | (position statement* or policy statement* or practice parameter* or best practice*).ti,ab,kf. |
| 14 | (standards or guideline or guidelines).ti,kf. |
| 15 | ((practice or treatment* or clinical) adj guideline*).ab. |
| 16 | (CPG or CPGs).ti. |
| 17 | consensus*.ti,kf. |
| 18 | consensus*.ab. /freq=2 |
| 19 | ((critical or clinical or practice) adj2 (path or paths or pathway or pathways or protocol*)).ti,ab,kf. |
| 20 | recommendat*.ti,kw. or guideline recommendation*.ab. |
| 21 | (care adj2 (standard or path or paths or pathway or pathways or map or maps or plan or plans)).ti,ab,kf. |
| 22 | (algorithm* adj2 (screening or examination or test or tested or testing or assessment* or diagnosis or diagnoses or diagnosed or diagnosing)).ti,ab,kf. |
| 23 | (algorithm* adj2 (pharmacotherap* or chemotherap* or chemotreatment* or therap* or treatment* or intervention*)).ti,ab,kf. |
| 24 | (guideline* or standards or consensus* or recommendat*).au. |
| 25 | (guideline* or standards or consensus* or recommendat*).co. |
| 26 | or/1-24 |
| 27 | exp *vitamin D/ or exp *vitamin D deficiency/ or ("vitamin d*" or "vitamin d2" or "vitamin d3" or "hydroxyvitamin d*" or "hydroxy-vitamin d*" or "hydroxylvitamin d*" or alfacalcidol* or alphacalcidol* or calcamine or calciferol* or calciol* or calcidiol* or calcitriol* or calcifediol* or calciferol* or calcipotriene or calcipotriol* or cholecalciferol* or colecalcifer* or dehydrocholesterol* or "dihydroxyvitamin d*" or "dihydroxy-vitamin d*" or dihydroxycholecalciferol* or "dihydroxy-cholecalciferol*" or dihydroxycalciferol* or "dihydroxy-calciferol*" or dihydroxycolecalciferol* or dihydroxyergocalciferol* or "dihydroxy-ergocalciferol*" or doxercalciferol* or dihydrotachysterol* or dihydroercalciol* or dihydrotachysterin or doxercalciferol* or epicalcitriol* or ercalcidiol* or ercalcitriol* or ergocalciferol* or hydroxycholecalciferol* or hydroxycalciferol* or hydroxycolecalciferol or hydroxyergocalciferol* or oxacalcitriol* or oxavitamin or paricalcitol* or seocalcitol or tacalcitol or "d hypovitaminosis" or "hypo-vitaminos* d" or "hypovitaminosis d" or "d vitamin deficien*" or "vit* d deficien*" or "avitaminosis D" or "D avitaminosis" or ((nutri* or diet*) adj supplement*)).ti,ab. |
| 28 | 26 and 27 |
| 29 | 28 not (exp juvenile/ not exp adult/) not ((infant* or child* or newborn* or pediatric* or paediatric* or juvenile) not adult).ti. not ((exp animal/ or nonhuman/) not exp human/) not (letter or note or editorial or conference).pt. |
| 30 | limit 29 to dc=20130101-20241231 |

**Cochrane Library – Cochrane Reviews**

ID Search Hits

#1 ("vitamin d" OR "vitamin d2" OR "vitamin d3" OR "hydroxyvitamin d" OR "hydroxy-vitamin d" OR "hydroxylvitamin d" OR alfacalcidol* OR alphacalcidol* OR calcamine OR calciferol* OR calciol* OR calcidiol* OR calcitriol* OR calcifediol* OR calciferol* OR calcipotriene OR calcipotriol* OR cholecalciferol* OR colecalcifer* OR dehydrocholesterol* OR dihydroxyvitamin d* OR dihydroxy-vitamin d* OR dihydroxycholecalciferol* OR dihydroxy-cholecalciferol* OR dihydroxycalciferol* OR dihydroxy calciferol* OR dihydroxycolecalciferol* OR dihydroxyergocalciferol* OR dihydroxy ergocalciferol* OR doxercalciferol* OR dihydrotachysterol* OR dihydroercalciol* OR dihydrotachysterin OR doxercalciferol* OR epicalcitriol* OR ercalcidiol* OR ercalcitriol* OR ergocalciferol* OR hydroxycholecalciferol* OR hydroxycalciferol* OR hydroxycolecalciferol* OR hydroxyergocalciferol* OR oxacalcitriol* OR oxavitamin* OR paricalcitol* OR seocalcitol OR tacalcitol OR "d hypovitaminosis" OR "hypo-vitaminosis d" OR "hypovitaminosis d" OR "d vitamin deficiency" OR "vitamin d deficiency" OR "avitaminosis D" OR "D avitaminosis" OR ((nutri* OR diet*) NEAR/3 supplement*) ):ti,ab

#2 (standards OR guideline* OR recommendat*):ti

#3 (position NEXT statement* OR policy NEXT statement* OR practice NEXT parameter* OR best NEXT practice* OR practice guideline* OR treatment guideline* OR CPG OR CPGs OR clinical NEXT guideline* OR guideline NEXT recommendation* OR consensus* OR ((critical OR clinical OR practice) AND (path OR paths OR pathway OR pathways OR protocol* OR bulletin OR bulletins)) OR (care AND (standard OR path OR paths OR pathway OR pathways OR map OR maps OR plan OR plans)) OR (algorithm* AND (screening OR examination OR test OR tested OR testing OR assessment* OR diagnosis OR diagnoses OR diagnosed OR diagnosing)) OR (algorithm* AND (pharmacotherap* OR chemotherap* OR chemotreatment* OR therap* OR treatment* OR intervention*))):ti,ab

#4 #2 OR #3

#5 #1 AND #4

#6 #5 NOT ((infant* OR child* OR newborn* OR pediatric* OR paediatric* OR juvenile*) NOT adult*):ti

Limits: Date range: 01/01/2013 to 31/12/2024

**Cochrane Reviews only**

**Google Scholar (first 200 results** sorted by relevance out of 23’600, date range 2013-2024)

"vitamin d"|"hydroxyvitamin d"|alfacalcidol|colecalciferol|cholecalciferol|calciferol|calcitriol guideline|guidelines|"position statement"|consensus|recommendations deficiency|malnutrition|screening|supplementation|prescription|prevention -pediatric

Complementary searches:

**Alert Service**A search alert was set in Google Scholar and followed to ensure no eligible articles were published and eventually missed.

**Reference lists** of included studies were hand-searched.

**Google search**
"vitamin d" guideline|guidelines|"position statement"|consensus|recommendations deficiency|malnutrition|screening|supplementation|prevention|prescription adult|adults

NICE <https://www.guidelines.co.uk/> OR <https://www.nice.org.uk/guidance>

**Table 2**

Overview of included guidelines (Full analysis)

| **Guideline name** | **Country/**  **Year** | **Screening recommended** | **Treatment recommended** | **Purpose of supplemen-**  **tation** | **25-OH-D threshold**  **[nmol/l]** | **Conclusion /Recommendations** | **Gaps and Research Needs** |
| --- | --- | --- | --- | --- | --- | --- | --- |
| Vitamin D supplementation in elderly or postmenopausal women: a 2013 update of the 2008 recommendations, European society for clinical and economic aspects of osteoporosis and osteoarthritis (ESCEO) | Europe, 2013 | Yes: bone diseases, old age, dark skin, obesity, pregnancy/lacating, sports, chronic kidney disease, liver failure, malabsorption syndromes, medication (antiepileptic drugs, glucocorticoids, AIDS drugs, antifungals, cholestyramine) and granulomaforming diseases | Elderly, postmenopausal women and osteoporotic patients with <50 nmol/L treated: between 800 and 1000 IU/day of vitamin D. A serum 25-(OH) D level of 75 nmol/L is treatment threshold in fragile elderly patients who are at particular risk of falls and fracture | Prevention of osteoporosis | 50 – 75 | The ESCEO recommends that 50 nmol/L should be the minimal serum 25(OH)D concentration at the population level and in patients with osteoporosis to ensure optimal bone health | Above the threshold of 50 nmol/L, there is no clear evidence for additional benefits of supplementation |
| Vitamin D - a systematic literature review for the 5th edition of the Nordic Nutrition Recommendations, Nordic Nutrition | Finland, Norway, Iceland, 2013 | No recommendation | 800 IU/day | bone health, reduction of total mortality, and the risk of falling | 50 | Data shows that 25(OH)D concentration of 50 nmol/l would reflect a sufficient vitamin D status. | There are large differences in results depending on assay methods/ laboratories measuring 25(OH)D. Moreover, the dose-response of vitamin D on serum 25(OH)D is not well established and is dependent on the basal concentrations, sunshine exposure, and dietary intake. |
| Recommendations Abstracted from the American Geriatrics Society Consensus Statement on Vitamin D for Prevention of Falls and Their Consequences, American geriatrics | USA, 2014 | Yes: obesity, malabsorption syndromes, individuals taking medications that bind vitamin D in the gut or accelerate the breakdown of vitamin D (e.g., cholestyramine inducers of the cytochrome P450 pathway such as phenytoin and phenobarbital). | All adults ≥ 65y: 1000UI/d | Reduce falls | 75 | The workgroup concluded that the goal to reduce fall injuries related to low vitamin D status could be achieved safely and would not require practitioners to measure serum 25(OH)D | Not specified |
| Celiac disease: diagnosis and treatment, Danish Society for Gastroenterology | Denmark, 2014 | Yes: All with untreated celiac disease | Levels should be monitored until normalisation. If normalisation of plasma levels is not obtained by oral treatment, parenteral administration should be considered | Prevention of osteopenia/porosis and deficiencies | Not specified | The monitoring of antibody levels and malabsorption markers is crucial during follow-up and allows for early treatment of disease complications. | Not specified |
| European Society of Endocrinology clinical guideline: Treatment of chronic hypoparathyroidism in adults | Europe, 2015 | Not specified but recommend adequate vitamin D status: Indirectly, all with chronic hypoparathyroidism | Vitamin D supplementations in a daily dose of 400– 800 IU to patients treated with activated vitamin D analogues | Reduce urinary calcium, risk of extra skeletal calcifications and symptoms of hypocalcemia | **>**50 | This guideline is mainly based on how patients are managed in clinical practice, as reported in small case series, and based on the experiences of the authors. | Little evidence is available on how best to treat hypoparathyroidism. No studies are available relating target calcium levels with clinically relevant endpoints. Hence it is not possible to formulate recommendations based on strict evidence. |
| Lack of Evidence linking Calcium with or without Vitamin D Supplementation to Cardiovascular Disease in generally healthy Adults: A clinical Guideline from the National Osteoporosis Foundation and the American Society for Preventive Cardiology | USA, 2016 | Not recommended | Not specified | Decrease all-cause mortality and risk of cardiovascular disease | Not specified | There is moderate-quality evidence that calcium with or without vitamin D intake has no relationship (beneficial or harmful) to the risk for cardiovascular and cerebrovascular disease, mortality, or all-cause mortality in generally healthy adults. | Not specified |
| Vitamin D: supplement use in specific population groups, NICE | UK, 2017 | Yes: only if someone has symptoms of deficiency or is at very high risk (low exposure to sunlight, osteomalacia or have had a fall). | Treatment recommended in specific groups: infants and children (<4y.); pregnant and breastfeeding women, particularly teenagers and young women; people over 65; people who have low or no exposure to the sun; people with dark skin; people with particular diet (avoid nuts, are vegan or have a halal or kosher diet). Supplements should undergo quality control checks to ensure they contain the correct dose of vitamin D. | Not defined, “good health” | <25 | This guideline aims to prevent vitamin D deficiency in specific populations | Cost-effectiveness analyses, monitoring supplementation |
| Treatment of low bone density or osteoporosis to prevent fractures in men and women: A clinical practice guideline update from the American college of physicians | USA, 2017 | Not specified | Osteoporosis treatment regimens and the effectiveness of these regimens on fracture prevention is unclear. Most trials with bisphosphonate therapy gave women calcium and vitamin D supplements. | Fractures and adverse events | Not specified | Treatment of low bone density or osteoporosis to prevent fractures | Most of the evidence for treating osteoporotic men is based on trials that included women, and further research is needed on the treatment of men. Studies directly addressing the efficacy of pharmacologic treatments for reducing fractures in patients with osteopenia are also needed. |
| Diagnosis, Evaluation, Prevention, and Treatment of Chronic Kidney Disease-Mineral and Bone Disorder: Synopsis of the kidney disease: Improving Global Outcomes 2017 Clinical Practice Guideline Update, KDIGO | International, 2018 | Yes: Only patients with levels of intact PTH progressively rising or persistently above the upper normal limit should be evaluated for vitamin d deficiency | In patients with CKD G5D requiring PTH lowering therapy, it is suggested calcimimetics, calcitriol, or vitamin D analogues, or a combination of calcimimetics with calcitriol or vitamin D analogues | Prevention and treatment of secondary hyperpara-thyroidism | Not specified | Calcimimetics, calcitriol, and vitamin D analogues are acceptable first-line options in patients receiving dialysis. Patients with CKD G3a to G5 not on dialysis, it is suggested that calcitriol and vitamin D analogues not be used routinely. Otherwise, there is an increased risk of hypercalcemia. | Studies with patient centered outcomes and surrogate end points are needed to determine the benefits and risks of treatment with calcitriol and vitamin D in patients with CKD G3a – 5and mild or severe SHPT.  Small number of recommendations. |
| Vitamin D, calcium, or combined supplementation for the primary prevention of fractures in community-dwelling adults, recommendation statement, US Preventive Services Task Force | USA, 2018 | Not specified | The USPSTF recommends against daily supplementation with 400 IU or less of vitamin D and 1000 mg or less of calcium for the primary prevention of fractures in community-dwelling, postmenopausal women. | Primary Prevention of Fractures | Not specified | The USPSTF concludes that the current evidence is insufficient to assess the balance of the benefits and harms of vitamin D and calcium supplementation, alone or combined, for the primary prevention of fractures in community-dwelling, asymptomatic men, and premenopausal women. The treatment of vitamin D is not recommended in primary prevention of fractures in community-dwelling, postmenopausal women. | Daily supplementation with doses greater than 400 IU of vitamin D and greater than 1000 mg of calcium reduces fracture incidence in postmenopausal women and in older men. |
| Current Vitamin D status in European and Middle East countries and strategies to prevent Vitamin D deficiency: a position statement of the European Calcified Tissue Society | Europe, 2019 | Yes: Individuals at risk for deficiency | Vitamin D supplements are recommended for special risk groups to increase the serum 25(OH)D concentration above 50 nmol/L in all countries of Europe and the Middle Eastern. Vitamin D supplement (400-800 IU/d) is advised to all older institutionalized subjects and should be considered for all older persons above 70y. | Bone health | 50 | According to current evidence, the desirable serum 25(OH)D concentration is set at 50 nmol/L or higher. | Future research should include genetic studies to better define individual vulnerability for vitamin D deficiency, and Mendelian randomization studies to address the effect of vitamin D deficiency on long-term non-skeletal outcomes such as cancer. |
| British Society of Gastroenterology consensus guidelines on the management of inflammatory bowel disease in adults | UK, 2019 | Yes: Vitamin D levels should be measured, and deficiency corrected in Crohn’s disease and ulcerative colitis | It is recommended that all patients receiving a course of corticosteroids for a disease flare should receive an intake of 800-1000 mg/d calcium and 800  IU/day vitamin D. | Deficiency, correction, prevention of osteoporosis, glucocorticoid use | Not specified | Patients with Crohn’s disease showed an increase in bone density with a daily intake of 800 IU vitamin D over 4 years. | Not specified |
| Clinical practice recommendations for the diagnosis and management of X-linked hypophosphatemia | International, 2019 | Yes: Children and adults with X-linked hypophosphatemia (XLH) and any first-generation family member of a patient with XLH should be investigated | Dosage should be adjusted to keep PTH levels within the normal range (10-65pg/ml); range for Calcitriol 0.5-0.75mcg/d | Prevention of calcitriol deficiency, hyperparathyroidism, increase phosphate absorption from the gut, improve the dentin mineralization and reduce dental abscesses and severity of peridontitis | No specific value for insufficiency specified. Dose can be adjusted based on serum levels of PTH and urinary calcium excretion. | XLH should be corrected by supplementation with native vitamin D in children. Optimal dose varies from patient to patient. During pregnancy 25(OH) vitamin d levels should be monitored and adjusted. | Not specific gaps or research needs for vitamin D mentioned |
| Vitamin D and bone health: a practical clinical guideline for patient management, Royal Osteoporosis Society | Europe, 2020 | Yes: Only in patients with symptoms of bone diseases who benefit from vitamin D supplementation (Rickets, Osteomalacia, Symptomatic hypocalcemia, patients with musculoskeletal symptoms) | <30nmol/l): high dose treatment initially, then long-term maintenance treatment required including lifestyle and diet advice.  30-50 nmol/l: over the counter dose of 800– 2000UI/d, long term. Diet and lifestyle advice. People aged 65 years and over and people who are not exposed to much sun: 1000 mg/d calcium and 400UI/d vitamin D. Osteoporosis: 1000 mg/d calcium and 400UI/d vit. D. Osteomalacia: 400UI/d of vit. D with calcium if needed. All pregnant and breastfeeding women: 400UI/d | Overall Health | 50 | For most patients, as there are no convincing benefits that are likely to outweigh potential risks, lower dose of vitamin D (400 IU/d) schemes are recommended. | Not specified |
| Vitamin D testing, Swiss Federal Office of Public Health (FOPH) | Switzerland, 2020 | Yes: risk groups | Supplementation with a dose of 600 to 800 IU/d vitamin D is recommended for adults | Overall Health | 50 | No sufficient evidence that would support the utility to test vitamin D in persons with bone disorders, older adults, obese individuals, pregnant women, people with dark skin, and athletes. | Not specified |
| American Association of Clinical Endocrinologists/ American College of Endocrinology Clinical Practice Guidelines for the Diagnosis and Treatment of Postmenopausal Osteoporosis-Update | USA, 2020 | Yes: Risk for vitamin D insufficiency, particularly those with osteoporosis and 25(OH)D levels <75 nmol/L. | Osteoporosis with serum level below 75 nmol/L. Vitamin D3 1000- 2000 IU/d | May increase response to bisphosphonate therapy, increase  BMD and prevent fractures | 30-50 | The effectiveness of anti-osteoporosis treatment can be improved when serum optimal 25(OH)D levels are reached in patients with osteoporosis risk. | More study is needed to determine the most effective means of communicating benefit and risk in the management of osteoporosis |
| The Belgian Bone Club 2020 guidelines for the management of osteoporosis in postmenopausal women | Belgia, 2020 | Yes: Postmenopausal women with at least one major risk factor, and who therefore should undergo further assessment of osteoporosis | In patients who receive antiresorptive or anabolic drugs (independent of vitamin D status) to reduce the risk of hypocalcaemia: 800−1000 IU/d. | Reduce fracture risk and risk of hypocalcaemia if treated with antiresorptive or anabolic drugs | 50 | In patients who receive antiresorptive or anabolic drugs Vitamin D of 800-1000 IU/d with a monitoring of the 25(OH)D level was recommended. High doses (e.g., 250,000–1,000,000 IU every 3– 12 months) led to a higher risk of falls and fractures in older patients. | Not specified |
| Executive Summary of the Academy of Nutrition and Dietetics and the National Kidney Foundation Clinical Practice Guideline for Nutrition in CKD | USA, 2020 | Not specified | Patients with CKD 1-5, CKD post transplantation and CKD 1-5 with nephrotic range and proteinuria, it is reasonable to consider supplementation of cholecalciferol, ergocalciferol, or other safe and effective 25(OH)D precursors | Correct deficiency, maintain calcium homeostasis | Not specified | In adults with CKD 1-5 and CKD post transplantation, it is suggested prescribing vitamin D supplementation in the form of cholecalciferol or ergocalciferol to correct 25(OH)D deficiency/insufficiency. | Not specified |
| Recommendations Based on Evidence by the Andalusian Group for Nutrition Reflection and Investigation (GARIN) for the Pre- and Postoperative Management of Patients undergoing Obesity Surgery | Europe, 2020 | Yes: All patients undergoing bariatric surgery should be screened preoperatively and treated accordingly | Preoperatively with a goal of > 50 nmol/L of 25(OH)D levels, postoperatively: 880 IU/d of cholecalciferol/d. In biliopancreatic diversion/Scopinaro surgeries the GARIN group recommends a higher intake of calcium (2000 mg/d) and especially a higher intake of vitamin D (2000 IU/d). | Pleiotropic effects (prevention of elevated bone turnover, SHPT and fractures) | 50 | The postoperative use of calcium (1000 mg/d) and vitamin D (880 IU of cholecalciferol) supplements are recommended. | Not specified |
| Osteoporosis clinical guideline for prevention and treatment executive summary, National Osteoporosis Guideline Group | Europe, 2021 | Yes: suggested for the investigation of osteoporosis/fragility fractures | Postmenopausal women and men ≥50y with osteoporosis or who are at risk of fragility.  Fractures are recommended: daily dose of 800IU/d cholecalciferol | Prevention of osteoporosis | Not specified | Not specified | Not specified |
| Screening for Vitamin D, US Preventive Service Task Force | USA, 2021 | Not recommended. Current evidence is inadequate to determine whether screening or treatment of asymptomatic low vitamin d levels improve clinical outcomes in community-dwelling adults. | People with low vitamin D | Overall Health | Not specified | The evidence was insufficient to assess the balance of benefits and harms of screening for vitamin D deficiency in asymptomatic adults. | Different studies using different cutoffs to define a low vitamin d level, or the cutoff may vary by individual or by subpopulation. More research is needed to define the cutoffs of vitamin d deficiency and studies on the benefits and harms of screening. |
| Congress of Neurological Surgeons Systematic Review and Evidence-Based Guidelines for Perioperative Spine: Preoperative Osteoporosis Assessment | USA, 2021 | Yes: preoperative osteoporosis assessment | Patients with osteoporosis. Daily dose not specified | Increased risk of postoperative osteoporosis related adverse effects | 50 | Information about preoperative assessment and risk of postoperative adverse events for patients with osteoporosis is important | The lack of Level I evidence is an area for improvement that would also benefit future guidelines. Future research should include randomized controlled studies to compare the efficacy of preoperative osteoporosis treatment protocols (single or multiagent), such as vitamin D3 |
| Chronic Obstructive Pulmonary Disease: A 2019 Evidence Analysis Centre Evidence- Based Practice, The academy of Nutrition and dietetics | USA, 2021 | Yes: COPD patients | If insufficient or deficient, vitamin D supplementation should be considered. Vitamin D should be administered according to local protocol or national clinical guidelines and clinical expertise | Reduce Exacerbations | 25 nmol/L or below, supplementation is recommended to optimize serum 25(OH)D status; >25 nmol/L can be considered | Evidence from adults with COPD with serum 25(OH)D levels <25nmol/L showed that vitamin D supplementation decreased exacerbations. While vitamin D is important for general health, vitamin D supplementation in those with serum 25(OH)D levels 25- 72.5 nmol/L may or may not improve lung function or reduce exacerbations in adults with COPD | The role for nutrition in the management of patients with COPD, and future high-quality research is needed to define optimal treatment strategies. |
| Vitamin, Mineral, and Multivitamin Supplementation to Prevent Cardiovascular Disease and Cancer, US Preventive Services Task Force Recommendation Statement | USA, 2022 | Not recommended | Not recommended | Decrease mortality of cardiovascular disease or cancer | Not specified | There was found no difference in all-cause mortality associated with vitamin D use. Vitamin D supplementation was not associated with any difference in cancer mortality or cancer incidence. | It is unclear whether the effect of vitamin D on health outcomes might vary based on patient population characteristics or an unidentified factor. The trials that reported on cancer mortality ranged from 3.3 to 7 years of follow-up. Studies are needed that provide whether vitamin D supplementation has an effect on cancer mortality. |
| Guideline No. 422g: Menopause and Osteoporosis, Society of Gynaecologists of Canada | Canada, 2022 | Yes: all adults >65y and all postmenopausal women | All patients with postmenopausal osteoporosis,  800-2000IU/d | Reduce fracture risk | 75-125 | Calcium-enriched diet with adequate vitamin D supplementation is advised | Careful patient evaluation, with exclusion of secondary causes of osteoporosis, should be followed by assessment of fracture risk. |
| Definition, Assessment, and Management of Vitamin D Inadequacy: Suggestions, Recommendations, and Warnings from the Italian Society for Osteoporosis, Mineral Metabolism and Bone Diseases (SIOMMMS) | Italy, 2022 | Yes: all patients at risk (>75y, inadequate solar exposure, Obesity, Pregnancy and breast-feeding, metabolic bone diseases and other skeletal disorders, vegan diet, Anorexia nervosa, Chronic renal failure, Cancer, Type 2 diabetes mellitus, intestinal malabsorption and bariatric surgery, Cystic fibrosis, treated with drugs that interfere with the absorption or hepatic metabolism of vitamin D)  Not recommended in adult general population | Vitamin D supplementation with 800-2000IU/d together with adequate calcium intake 800-1000mg/d | Reduce the risk of fractures | 50-125 in general population; 75-125 in population at risk | Screening and treatment of all patients at risk, supplementation with a regimen able to maintain optimal vitamin D levels should be provided until the cause of vitamin D deficiency has been removed | There is still no evidence that screening the general population and the consequent treatment of vitamin D deficient cases would represent a cost-effective procedure. |
| Evaluation and Management of Hypoparathyroidism Summary Statement and Guidelines from the Second International Workshop | International, 2022 | Yes: all patients with Hypoparathyroidism | Treat with calcium and an active vitamin D analogue, with the goal of raising serum calcium to the target range. Avoid hypercalciuria when titrating calcium and active vitamin D analogue therapy, aiming for low normal plasma calcium levels.  Cholecalciferol: 1000-100000IU/d based on 25(OH)D level  Calcitriol:  0.25-3mcg/d administered in divided doses | Decrease morbidity of Hypoparathyroidism | 75-125 | Early identification of HypoPT with initiation of effective conventional therapy with calcium and active vitamin D metabolites is helpful in improving symptoms. | Future studies on the complications of HypoPT, including cardiovascular disease, cataracts, BGCs, infections, malignancy, and neuropsychiatric disorders, are needed. Future trials should include patients with genetic disorders to clarify best management. |
| 2022 American College of Rheumatology Guideline for the Prevention and Treatment of Glucocorticoid-Induced Osteoporosis | USA, 2023 | Yes: patients >40y with GC treatment for >3months | Adequate age-appropriate dietary and supplemental intake of calcium and vitamin D, weight-bearing exercise, and avoidance of smoking and alcohol intake is encouraged for all patients receiving GC.  Vitamin D 600-800UI/d or more is typically required. | Reduce fractures and their adverse consequences, while minimizing harm due to medications | 75-125 | For adults at medium, high, or very high fracture risk, we strongly recommend pharmacologic treatment. | The evidence for calcium and vitamin D supplementation for fracture reduction in GIOP is low to very low. |
| Vitamin D status and supplementation before and after Bariatric Surgery: Recommendations based on a systematic review and meta-analysis | International, 2023 | Yes: all bariatric patients, pre- and post-surgery | All candidates for bariatric surgery with vitamin D deficiency should be supplemented accordingly.  Post-surgery patients: high dose supplementation with 2000IU/d and periodically monitoring of 25(OH) D levels is recommended | Skeletal health | 75 | High-dose supplementation is recommended in patients after bariatric surgery. | Evidence levels were influenced by inconsistently controlled nature of the studies currently available in the literature and included in the meta-analysis, mostly conducted with only observational and retrospective designs, and evaluating non-randomised patient cohorts. |
| 2023 AHA/ACC/ACCP/ASPC/NLA/PCNA Guideline for the Management of Patients with Chronic Coronary Disease: A Report of the American Heart Association / American College of Cardiology Joint Committee on Clinical Practice Guidelines | USA, 2023 | Not recommended | Not recommended | Not specified | Not specified | In patients with CCD, the use of nonprescription or dietary supplements, including vitamin D and calcium, is not beneficial to reduce the risk of acute CVD events. | Despite observational studies, insufficient evidence is available that shows vitamin D supplementation reduces CVD events. |
| Vitamin D for the Prevention of Disease: An Endocrine Society Clinical Practice Guideline | International, 2024 | Not recommended | Recommended for children and adolescents aged 1 to 18 years (300-2000IU/day), general population >75 years (400-3333IU/day), pregnant women (600-5000IU/day), adults with high-risk prediabetes (842-7543IU/day) and adults >50 years who have indications for vitamin D supplementation or treatment (dosage not specified). Daily intake of fortified foods and sun exposure, lifestyle modification (prediabetes) are recommended. | Lower risk of general mortality, preeclampsia, intra-uterine mortality, preterm birth, small-for-gestational-age birth, neonatal mortality, and progression from prediabetes to diabetes in the general population | Not specified | The panel suggests empiric vitamin D for those aged 1 to 18 years and adults over 75 years of age, those who are pregnant, and those with high-risk prediabetes (general population). The panel suggests against routine 25(OH)D testing in the absence of established indications. The Endocrine Society no longer endorses specific 25(OH)D levels to define vitamin D sufficiency, insufficiency and deficiency. | Further research is needed to determine optimal 25(OH)D levels for specific health benefits. No trials were designed or powered to address the effect of vitamin D in subgroups stratified by either baseline or achieved 25(OH)D levels. Many trials were considered to be of insufficient duration to adequately assess the effect of the vitamin D intervention on some outcomes, due to the long latency for the development of chronic diseases. The trials were performed in overall healthy populations at average risk for the outcomes of interest, therefore the recommendations are limited to generally healthy individuals without established indications for vitamin D treatment or 25(OH) D testing. In most trials, study participants were largely of European ancestry or identified as non-Hispanic White. |

**Table 3**

Overview of excluded guidelines

| **Guideline name** | **Reason for exclusion** | **Year of publication** | **Country** | **Institution introducing the guideline** |
| --- | --- | --- | --- | --- |
| Diagnosis and management of osteoporosis in postmenopausal women and older men in the UK:  National Osteoporosis Guideline  Group (NOGG) update 2013 | In guideline 2016 included. | 2013 | NOGG | UK |
| Vitamin D and calcium supplementation to prevent fractures in adults: U.S. preventive services task Force recommendation statement | Newer guideline available. | 2013 | US Preventive Task Force | US |
| Recommendations Abstracted from the American Geriatrics Society Consensus Statement on Vitamin D  for Prevention of Falls and Their Consequences | Consensus Statement | 2014 | American Geriatrics Society | US |
| National Osteoporosis Society Vitamin D Guideline Summary | Commentary of IOM guidelines 2010. | 2014 | National Osteoporosis Society | UK |
| Vitamin D and cognition in older  adults: updated international recommendations | No Systemic Review (SR), only Delphi. | 2014 | International | International |
| Vitamin D and multiple sclerosis | No guideline/Narrative Review. | 2013 | MS Society UK | UK |
| The role of dietary protein and vitamin D in maintaining musculoskeletal health in postmenopausal women | Same guideline as published in 2013. | 2014 | European Society for Clinical and Economic Aspects of Osteoporosis and  Osteoarthritis | UK |
| Emerging Practice Concerning Vitamin D in Primary Care | Qualitative study, no guidelines. | 2014 | Primary Care | UK |
| Vitamin, mineral, and multivitamin supplements for the primary prevention of cardiovascular disease and cancer: U.S. Preventive services Task Force recommendation statement | Not specific vitamin D recommendation. | 2014 | US Preventive Task Force | US |
| Nutritional guidelines for older people in Finland | No Systemic Review | 2014 | National Nutritional Council in Finland. | Finland |
| Vitamin D supplementation review and recommendations for women diagnosed with breast or ovary cancer in the context of bone health and cancer prognosis/risk | Review and recommendation. | 2015 | Department of  Medical Oncology,  Hospital La Zarzuela | Spain |
| Recommended intakes of Vitamin D to optimise health, associated circulating 25-hydroxyVitamin D concentrations, and dosing regimens to treat deficiency: Workshop report and overview of current literature | No Systematic Review, only Workshop. | 2015 | NutriProfiel | Denmark |
| ACG clinical guideline: Primary sclerosing cholangitis | No Systematic Review | 2015 | American College of Gastroenterology | US |
| Recommended European Society of Parenteral and Enteral Nutrition protein and energy intakes and weight loss in patients with head and neck cancer | Study on patients, not guidelines. | 2016 | European Society of Parenteral and Enteral Nutrition | Europe |
| Lifestyle and Dietary Interventions in the Management of Non-alcoholic Fatty Liver Disease | No Vitamin D recommendation. | 2016 |  | US |
| Vitamin D in patients with chronic kidney disease: a position statement of the Working Group “Trace Elements and Mineral Metabolism” of the Italian Society of Nephrology | Position statement | 2016 |  | International |
| Serum vitamin D status in adult patients with atopic dermatitis: Recommendations for daily practice | Study on patients, not guidelines. | 2016 | the American Academy of Dermatology | US |
| Non-pharmacological interventions to prevent or treat pressure ulcers in older patients: Clinical practice recommendations. The SENATORONTOP series | No Vitamin D recommendation. | 2016 |  | UK |
| Guidelines on Vitamin D replacement in bariatric surgery: Identification and systematic appraisal | Review of guidelines. | 2016 | Clinical Practice Guidelines | US |
| Glucocorticoid-induced osteoporosis: pathophysiological role of  GH/IGF-I and PTH/VITAMIN D axes, treatment options and guidelines | Summary of a meeting. | 2016 | 9th GlucocorticoidInduced Osteoporosis Meeting | Europe |
| Chronic kidney disease-mineral and bone disorder: Guidelines for diagnosis, treatment, and management | Commentary/Narrative Review. | 2016 | JAAPA | US |
| World Allergy Organization-McMaster University Guidelines for Allergic Disease Prevention (GLAD-P): Vitamin D | Pregnant, breastfeeding, infants | 2016 | World Allergy Organization (WAO) | International |
| The American Association of Endocrine Surgeons guidelines for definitive management of primary hyperparathyroidism | No Systemic Review | 2016 | American Association of Endocrine Surgeons | US |
| American College of Foot and Ankle Surgeons: Clinical Consensus Statement: Perioperative Management | No Systemic Review | 2017 | The American College of Foot and Ankle Surgeons | US |
| Vitamin D Deficiency, Its Role in  Health and Disease, and Current Supplementation Recommendations | No Systemic Review | 2017 |  | US |
| Nutritional Recommendations for Adult Bariatric Surgery Patients:  Clinical Practice | No Systemic Review | 2017 | American Society for Nutrition | US |
| Management of bone health in patients with celiac disease: Practical guide for clinicians | Probably Narrative Review. | 2018 |  | Canada |
| Nutritional Intervention in Sarcopenia: Report from the International Conference on Frailty and Sarcopenia Research Task Force | No Vitamin D recommendation. | 2018 | Sarcopenia Research Task Force | International |
| IOC consensus statement: Dietary supplements and the high-performance athlete | No Systemic Review | 2018 |  | US |
| American Society for Enhanced recovery and perioperative quality initiative joint consensus statement on nutrition screening and therapy within a surgical enhanced recovery pathway | No Vitamin D recommendation. | 2018 | American Society for Enhanced Recovery and Perioperative Quality | US |
| Vitamin D, Calcium, or Combined  Supplementation for the Primary Prevention of Fractures in Community-Dwelling Older Adults: Recommendation Statement | Duplicate (published in diff. Journals). | 2018 | US Preventive Task Force | US |
| The Use of Multivitamin/Multimineral Supplements: A Modified Delphi Consensus Panel Report | No Systemic Review | 2018 | International | International |
| ACG clinical guideline: Alcoholic liver disease | No vitamin D recommendation. | 2018 | American College of Gastroenterology | US |
| Nutrition therapy and critical illness: practical guidance for the ICU, post-ICU, and long-term convalescence phases | No Vitamin D recommendation. | 2019 |  | Europe |
| VA/DoD Clinical Practice Guideline:  Diagnosis and Treatment of Low  Back Pain | No Vitamin D recommendation. | 2019 | VA/DoD | US |
| Systematic review of natural and miscellaneous agents for the management of oral mucositis in cancer patients and clinical practice guidelines-part 1: vitamins, minerals, and  nutritional supplements | No Vitamin D recommendation. | 2019 | Mucositis Study Group of the Multinational Association of Supportive Care in Cancer / International Society of Oral Oncology | International |
| Canadian recommendations for vitamin D intake for persons affected by multiple sclerosis | No Systemic Review | 2020 | Multiple Sclerosis (MS) Society of Canada | Canada |
| American Association of Clinical Endocrinologists/American College of Endocrinology Clinical Practice  Guidelines for the Diagnosis and Treatment of Postmenopausal Osteoporosis-2020 Update | No Systemic Review | 2020 | American College of Clinical Endocrinologists | US |
| Management of cancer cachexia: ASCO guideline | No Vitamin D recommendation. | 2020 | ASCO | US |
| The 2020 Updated KDOQI Clinical  Practice Guidelines for Nutrition in  Chronic Kidney Disease | Commentary | 2020 | Clinical Practice Guidelines for Nutrition in Chronic Kidney Disease | US |
| Diagnosis and management of pancreatic exocrine insufficiency (PEI) in primary care: consensus guidance of a Canadian expert panel | No Systemic Review |  |  | Canada |
| Secondary Fracture Prevention: Consensus Clinical Recommendations from a Multistakeholder Coalition | Probably Narrative Review. | 2020 | American Society for Bone and Mineral Research | US |
| Recommendations and guidance on nutritional supplementation in the liver transplant setting | Probably Narrative Review | 2021 |  | Spain |
| Preoperative Management of Surgical Patients Using Dietary Supplements: Society for Perioperative Assessment and Quality Improvement  (SPAQI) Consensus Statement | No Systemic Review | 2021 | Society for Perioperative Assessment and Quality Improvement | US |
| Management of osteoporosis in postmenopausal women: the 2021 position statement of The North American Menopause Society | No Systemic Review | 2021 | The North American Menopause Society | US |
| Prevention of Osteoporotic Fractures in Residential Aged Care: | Updated Consensus Recommendations. No  Systematic Review | 2022 |  | Australia |
| Nutrition in Thalassemia: A Systematic Review of Deficiency, Relations to Morbidity, and Supplementation Recommendations | Nutritional recommendation | 2022 |  | International |
| Epidemiology, natural course, and preventive measures of osteoporotic vertebral fractures: WFNS Spine Committee Recommendations | No Systemic Review | 2022 |  | US |
| **New exclusions 2024** |  |  |  |  |
| Polish guidelines for the diagnosis and management of osteoporosis: a review of 2014 update | Newer guideline available | 2014 | Poland | Polish association of orthopedics and osteoporosis |
| National Osteoporosis Society practical clinical guideline on vitamin D and bone health | Newer guideline available | 2015 | UK | National Osteoporosis Society |
| 2017 American College of Rheumatology Guideline for the Prevention and Treatment of Glucocorticoid-Induced Osteoporosis | Newer guideline available | 2017 | USA | American College of Rheumatology |
| Position statement: clinical management of vitamin D deficiency in adults | Position statement | 2018 | Italy | Italian Adssociation of Clinical Endocrinologists |
| Prevention and Treatment of Glucocorticoid-Induced Osteoporosis in Adults: Consensus Recommendations | No systematic review | 2022 | Belgia | Belgian Bone Club |
| French recommendations on the prevention and treatment of osteoporosis secondary to bariatric surgery | No systematic review | 2022 | France | Osteoporosis Research and Information Group, French Rheumatology Society |
| Clinical Practice in the Prevention, Diagnosis and Treatment of Vitamin D Deficiency: A Central and Eastern European Expert Consensus Statement | No systematic review | 2022 | Europe | Central and Eastern European Experts |
| Vitamin D in the older population: a consensus statement | No systematic review | 2022 | International | International Conference “Controversies in Vitamin D” |
| Guidelines for Preventing and Treating Vitamin D Deficiency: A 2023 Update in Poland | No systematic review | 2023 | Poland | Poland Experts |
| Consensus Statement on Vitamin D Status Assessment and Supplementation: Whys, Whens, and Hows | No systematic review | 2023 | International | International Conference “Controversies in Vitamin D” |
| AGA Clinical Practice Update on the Epidemiology, Evaluation, and Management of Exocrine Pancreatic Insufficiency: Expert Review | No systematic review | 2023 | USA | American Gastroenterological Association (AGA) |
| Management of Vertebral Fragility Fracture in Older People: Recommendations from a Spanish Consensus of Experts | No systematic review | 2024 | Spain | Spanish Experts |

**Table 4**

| **Guideline name** | **Year** | **Country** |
| --- | --- | --- |
| Vitamin D supplementation in elderly or postmenopausal women: a 2013 update of the 2008 recommendations, European society for clinical and economic aspects of osteoporosis and osteoarthritis (ESCEO) | 2013 | Switzerland, Belgia, Italy, UK, Germany |
| Vitamin D - a systematic literature review for the 5th edition of the Nordic Nutrition Recommendations, Nordic Nutrition | 2013 | Finland, Norway, Iceland |
| Celiac disease: diagnosis and treatment, Danish Society for Gastroenterology | 2014 | Denmark |
| European Society of Endocrinology clinical guideline: Treatment of chronic hypoparathyroidism in adults | 2015 | Norway, Denmark, Italy, Spain, Netherlands |
| Vitamin D: supplement use in specific population groups, NICE | 2017 | United Kingdom |
| Current Vitamin D status in European and Middle East countries and strategies to prevent Vitamin D deficiency: a position statement of the European Calcified Tissue Society | 2019 | Netherland, Ireland, Finland, Switzerland, Austria, Italy |
| British Society of Gastroenterology consensus guidelines on the management of inflammatory bowel disease in adults | 2019 | United Kingdom |
| Vitamin D and bone health: a practical clinical guideline for patient management, Royal Osteoporosis Society | 2020 | United Kingdom |
| Vitamin D testing, Swiss Federal Office of Public Health (FOPH) | 2020 | Switzerland |
| The Belgian Bone Club 2020 guidelines for the management of osteoporosis in postmenopausal women | 2020 | Belgia |
| Recommendations Based on Evidence by the Andalusian Group for Nutrition Reflection and Investigation (GARIN) for the Pre- and Postoperative Management of Patients undergoing Obesity Surgery | 2020 | Spain |
| Osteoporosis clinical guideline for prevention and treatment executive summary, National Osteoporosis Guideline Group | 2021 | United Kingdom |
| Definition, Assessment, and Management of Vitamin D Inadequacy: Suggestions, Recommendations, and Warnings from the Italian Society for Osteoporosis, Mineral Metabolism and Bone Diseases (SIOMMMS) | 2022 | Italy |
|  |  |  |
| Recommendations Abstracted from the American Geriatrics Society Consensus Statement on Vitamin D for Prevention of Falls and Their Consequences, American geriatrics | 2014 | USA |
| Lack of Evidence linking Calcium with or without Vitamin D Supplementation to Cardiovascular Disease in generally healthy Adults: A clinical Guideline from the National Osteoporosis Foundation and the American Society for Preventive Cardiology | 2016 | USA |
| Treatment of low bone density or osteoporosis to prevent fractures in men and women: A clinical practice guideline update from the American college of physicians | 2017 | USA |
| Vitamin D, calcium, or combined supplementation for the primary prevention of fractures in community-dwelling adults, recommendation statement, US Preventive Services Task Force | 2018 | USA |
| American Association of Clinical Endocrinologists/ American College of Endocrinology Clinical Practice Guidelines for the Diagnosis and Treatment of Postmenopausal Osteoporosis-Update | 2020 | USA |
| Executive Summary of the Academy of Nutrition and Dietetics and the National Kidney Foundation Clinical Practice Guideline for Nutrition in CKD | 2020 | USA |
| Screening for Vitamin D, US Preventive Service Task Force | 2021 | USA |
| Congress of Neurological Surgeons Systematic Review and Evidence-Based Guidelines for Perioperative Spine: Preoperative Osteoporosis Assessment | 2021 | USA |
| Chronic Obstructive Pulmonary Disease: A 2019 Evidence Analysis Centre Evidence- Based Practice, The academy of Nutrition and dietetics | 2021 | USA |
| Vitamin, Mineral, and Multivitamin Supplementation to Prevent Cardiovascular Disease and Cancer, US Preventive Services Task Force Recommendation Statement | 2022 | USA |
| Guideline No. 422g: Menopause and Osteoporosis, Society of Gynaecologists of Canada | 2022 | Canada |
| 2022 American College of Rheumatology Guideline for the Prevention and Treatment of Glucocorticoid-Induced Osteoporosis | 2023 | USA |
| 2023 AHA/ACC/ACCP/ASPC/NLA/PCNA Guideline for the Management of Patients with Chronic Coronary Disease: A Report of the American Heart Association / American College of Cardiology Joint Committee on Clinical Practice Guidelines | 2023 | USA |
|  |  |  |
| Diagnosis, Evaluation, Prevention, and Treatment of Chronic Kidney Disease-Mineral and Bone Disorder: Synopsis of the kidney disease: Improving Global Outcomes 2017 Clinical Practice Guideline Update, KDIGO | 2018 | International |
| Clinical practice recommendations for the diagnosis and management of X-linked hypophosphatemia | 2019 | International |
| Evaluation and Management of Hypoparathyroidism Summary Statement and Guidelines from the Second International Workshop | 2022 | International |
| Vitamin D status and supplementation before and after Bariatric Surgery: Recommendations based on a systematic review and meta-analysis | 2023 | International |
| Vitamin D for the Prevention of Disease: An Endocrine Society Clinical Practice Guideline | 2024 | International |

**Table 5**

Results on quality

| **Guideline name** | **Scope and Purpose** | **Stakeholder Involvement** | **Rigour of Developement** | **Clarity of Presentation** | **Applicability** | **Editorial Independence** | **Overall quality** |
| --- | --- | --- | --- | --- | --- | --- | --- |
| Vitamin D supplementation in elderly or postmenopausal women: a 2013 update of the 2008 recommendations, European society for clinical and economic aspects of osteoporosis and osteoarthritis (ESCEO) | 19/21 | 14/21 | 37/56 | 18/21 | 22/28 | 8/14 | 5/7 |
| Vitamin D - a systematic literature review for the 5th edition of the Nordic Nutrition Recommendations, Nordic Nutrition | 15/21 | 12/21 | 38/56 | 17/21 | 22/28 | 7/14 | 5/7 |
| Recommendations Abstracted from the American Geriatrics Society Consensus Statement on Vitamin D for Prevention of Falls and Their Consequences, American geriatrics | 21/21 | 15/21 | 38/56 | 18/21 | 24/28 | 8/14 | 6/7 |
| Celiac disease: diagnosis and treatment, Danish Society for Gastroenterology | 21/21 | 14/21 | 36/56 | 13/21 | 16/28 | 8/14 | 4/7 |
| European Society of Endocrinology clinical guideline: Treatment of chronic hypoparathyroidism in adults | 20/21 | 14/21 | 36/56 | 15/21 | 23/28 | 9/14 | 5/7 |
| Lack of Evidence linking Calcium with or without Vitamin D Supplementation to Cardiovascular Disease in generally healthy Adults: A clinical Guideline from the National Osteoporosis Foundation and the American Society for Preventive Cardiology | 15/21 | 13/21 | 29/56 | 12/21 | 9/28 | 10/14 | 4/7 |
| Vitamin D: supplement use in specific population groups, NICE | 17/21 | 14/21 | 40/56 | 19/21 | 25/28 | 6/14 | 6/7 |
| Treatment of low bone density or osteoporosis to prevent fractures in men and women: A clinical practice guideline update from the American college of physicians | 17/21 | 12/21 | 38/56 | 13/21 | 19/21 | 6/14 | 4/7 |
| Diagnosis, Evaluation, Prevention, and Treatment of Chronic Kidney Disease-Mineral and Bone Disorder: Synopsis of the kidney disease: Improving Global Outcomes 2017 Clinical Practice Guideline Update, KDIGO | 20/21 | 17/21 | 41/56 | 16/21 | 24/28 | 8/14 | 6/7 |
| Vitamin D, calcium, or combined supplementation for the primary prevention of fractures in community-dwelling adults, recommendation statement, US Preventive Services Task Force | 21/21 | 15/21 | 41/56 | 16/21 | 19/28 | 6/14 | 5/7 |
| Current Vitamin D status in European and Middle East countries and strategies to prevent Vitamin D deficiency: a position statement of the European Calcified Tissue Society | 20/21 | 15/21 | 42/56 | 18/21 | 25/28 | 7/14 | 6/7 |
| British Society of Gastroenterology consensus guidelines on the management of inflammatory bowel disease in adults | 21/21 | 15/21 | 41/56 | 19/21 | 21/28 | 8/14 | 6/7 |
| Clinical practice recommendations for the diagnosis and management of X-linked hypophosphatemia | 21/21 | 15/21 | 42/56 | 20/21 | 26/28 | 8/14 | 6/7 |
| Vitamin D and bone health: a practical clinical guideline for patient management, Royal Osteoporosis Society | 21/21 | 16/21 | 42/56 | 21/21 | 25/28 | 8/14 | 6/7 |
| Vitamin D testing, Swiss Federal Office of Public Health (FOPH) | 18/21 | 15/21 | 40/56 | 20/21 | 24/28 | 8/14 | 6/7 |
| American Association of Clinical Endocrinologists/ American College of Endocrinology Clinical Practice Guidelines for the Diagnosis and Treatment of Postmenopausal Osteoporosis-Update | 21/21 | 15/21 | 41/56 | 19/21 | 24/28 | 8/14 | 6/7 |
| The Belgian Bone Club 2020 guidelines for the management of osteoporosis in postmenopausal women | 21/21 | 17/21 | 39/56 | 20/21 | 23/28 | 8/14 | 6/7 |
| Executive Summary of the Academy of Nutrition and Dietetics and the National Kidney Foundation Clinical Practice Guideline for Nutrition in CKD | 18/21 | 13/21 | 35/56 | 13/21 | 17/28 | 6/14 | 4/7 |
| Recommendations Based on Evidence by the Andalusian Group for Nutrition Reflection and Investigation (GARIN) for the Pre- and Postoperative Management of Patients undergoing Obesity Surgery | 21/21 | 16/21 | 40/56 | 20/21 | 25/28 | 8/14 | 6/7 |
| Osteoporosis clinical guideline for prevention and treatment executive summary, National Osteoporosis Guideline Group | 20/21 | 14/21 | 36/56 | 14/21 | 17/28 | 6/14 | 5/7 |
| Screening for Vitamin D, US Preventive Service Task Force | 16/21 | 13/21 | 38/56 | 15/21 | 22/28 | 6/14 | 5/7 |
| Congress of Neurological Surgeons Systematic Review and Evidence-Based Guidelines for Perioperative Spine: Preoperative Osteoporosis Assessment | 21/21 | 13/21 | 41/56 | 18/21 | 25/28 | 8/14 | 6/7 |
| Chronic Obstructive Pulmonary Disease: A 2019 Evidence Analysis Centre Evidence- Based Practice, The academy of Nutrition and dietetics | 21/21 | 14/21 | 42/56 | 18/21 | 24/28 | 7/14 | 6/7 |
| Vitamin, Mineral, and Multivitamin Supplementation to Prevent Cardiovascular Disease and Cancer, US Preventive Services Task Force Recommendation Statemen | 16/21 | 13/21 | 31/56 | 12/21 | 17/28 | 8/14 | 4/7 |
| Guideline No. 422g: Menopause and Osteoporosis, Society of Gynaecologists of Canada | 21/21 | 21/21 | 41/56 | 21/21 | 21/28 | 8/14 | 6/7 |
| Definition, Assessment, and Management of Vitamin D Inadequacy: Suggestions, Recommendations, and Warnings from the Italian Society for Osteoporosis, Mineral Metabolism and Bone Diseases (SIOMMMS) | 21/21 | 16/21 | 47/56 | 21/21 | 26/28 | 8/14 | 6/7 |
| Evaluation and Management of Hypoparathyroidism Summary Statement and Guidelines from the Second International Workshop | 17/21 | 14/21 | 33/56 | 16/21 | 18/28 | 8/14 | 5/7 |
| 2022 American College of Rheumatology Guideline for the Prevention and Treatment of Glucocorticoid-Induced Osteoporosis | 20/21 | 15/21 | 37/56 | 18/21 | 21/28 | 8/14 | 6/7 |
| Vitamin D status and supplementation before and after Bariatric Surgery: Recommendations based on a systematic review and meta-analysis | 19/21 | 14/21 | 33/56 | 15/21 | 21/28 | 8/14 | 5/7 |
| 2023 AHA/ACC/ACCP/ASPC/NLA/PCNA Guideline for the Management of Patients with Chronic Coronary Disease: A Report of the American Heart Association / American College of Cardiology Joint Committee on Clinical Practice Guidelines | 16/21 | 15/21 | 26/56 | 12/21 | 18/28 | 8/14 | 4/7 |
| Vitamin D for the Prevention of Disease: An Endocrine Society Clinical Practice Guideline | 20/21 | 16/21 | 41/56 | 19/21 | 22/28 | 8/14 | 6/7 |
